# Specific Intratumoral Microbiome Signatures in Human Glioblastoma and Meningioma: Evidence for a Gut–Brain Microbial Axis

**DOI:** 10.3390/ijms262311290

**Published:** 2025-11-22

**Authors:** Dalila Mehelleb, Abderezak Ghidouche, Simone Baldi, Ferhat Djoudi, Sara Bertorello, Leandro Di Gloria, Matteo Ramazzotti, Elena Niccolai, Menad Madaoui, Idir Takbou, Souhil Tliba, Amedeo Amedei

**Affiliations:** 1 University of Bejaia, Faculty of Natural and Life Sciences, Bejaia 06000, Algeria; dalila.mehelleb@univ-bejaia.dz (D.M.); ferhat.djoudi@univ-bejaia.dz (F.D.);; 2 University of Bejaia, Laboratory of Microbial Ecology, Bejaia 06000, Algeria; 3 University of Bejaia, Laboratory of Cancer Bioengineering, Bejaia 06000, Algeria; 4 Department of Experimental and Clinical Medicine, University of Florence, 50134 Florence, Italy; sara.bertorello@unifi.it (S.B.);; 5 Department of Biomedical, Experimental and Clinical Sciences “Mario Serio”, University of Florence, 50134 Florence, Italy; 6 Department of Neurosurgery, University Hospital Center of Bejaia, Bejaia 06000, Algeria; 7 Department of Neurosurgery, University Hospital Center of Blida, Blida 09000, Algeria

**Keywords:** brain tumor, glioblastoma, meningioma, gut–brain axis, gut microbiome, intratumoral microbiome

## Abstract

Brain tumors (BTs), including glioblastoma (GBM) and meningioma (MGM), contribute significantly to the global cancer burden. The microbiome has been implicated in carcinogenesis, yet its role in BTs remains underexplored. We performed 16S rRNA gene sequencing of the gut microbiota (GM) and intratumoral microbiome (ItM) from fresh tissue samples of 9 patients with GBM and 18 with MGM. 12 age- and sex-matched healthy controls (HCs) were also enrolled. GM profiling revealed reduced alpha diversity and distinct microbial communities in BT patients versus HCs. Notably, Verrucomicrobiota and Synergistaceae were enriched, while Lachnospiraceae, Peptostreptococcaceae, and *Muribacter* spp. were depleted. GBM patients showed reductions in Peptostreptococcaceae and the *Eubacterium hallii* group, while MGM patients had increased *Synergistia* and *Erysipelatoclostridium*. Compared with MGM, GBM patients were enriched in Peptostreptococcales–Tissierellales, *Coprobacillus*, and *Peptoniphilus* but depleted in *Weissella*. Venn analysis revealed 176 genera shared across groups with unique taxa distinguishing tumor patients and HCs. ItM profiling revealed enrichment of Proteobacteria, Actinomycetota, and Campylobacterota in GBM, while MGM contained higher levels of Bacillota and Bacteroidota. GBM tissues harbored *Burkholderia-Caballeronia-Paraburkholderia*, *Helicobacter*, and *Leifsonia*, whereas MGM tissues were dominated by *Bacteroides* and *Blautia*. Notably, stool and tumor samples shared 91 genera in GBM and 105 in MGM. This study provides novel insights by (i) characterizing ItM from fresh samples, (ii) comparing ItM profiles of GBM and MGM, (iii) linking GM and ItM within the same patients, and (iv) suggesting potential clinical implications for BT management.

## 1. Introduction

According to the International Agency for Research on Cancer (IARC), 20 million new cancer cases and 9.7 million cancer-related deaths were reported worldwide in 2022, with incidence projected to rise by 77% by 2050 [1]. Among all malignancies, brain and central nervous system (CNS) tumors rank 19th in incidence and 12th in mortality globally [2]. While classical cancer etiology has emphasized genetic predisposition, environmental exposures, and lifestyle factors, a growing body of evidence implicates the gut microbiome (GM) in both tumor initiation and progression. Notably, infection-associated cancers alone account for approximately 2.2 million cases globally, 13% of total incidence, underscoring the role of microorganisms in oncogenesis [3]. Groundbreaking work by Nejman et al. demonstrated that each cancer type harbors a unique intratumoral microbiome (ItM). In their analysis of 1526 tumor and adjacent healthy tissue samples across seven malignancies, distinct, cancer-type-specific bacterial signatures were identified [4]. In addition, our previous reports documented presence of an ItM in both breast and adrenocortical tumors [5,6]. Beyond local effects, microbes from remote niches such as the oral cavity, lungs, pancreas, and colon have also been implicated in carcinogenesis at distant sites, highlighting both direct and indirect roles of the microbiome in cancer development [7,8,9]. Traditionally, the brain has been considered a sterile, immune-privileged organ protected by the blood–brain barrier (BBB). However, studies of the gut–brain axis reveal that the GM is essential for CNS homeostasis: germ-free mice exhibit both immune dysregulation and impaired BBB integrity, defects reversible upon microbial colonization [10,11]. Alterations in GM composition have also been associated with a spectrum of CNS disorders, including: (i) neurodegenerative diseases such as Alzheimer’s disease (AD) [12], Parkinson’s disease (PD) [13], multiple sclerosis (MS) [14] and amyotrophic lateral sclerosis (ALS) [15,16]; (ii) neuropsychiatric conditions such as depression [17] and autism spectrum disorders (ASD) [18]; and (iii) cerebrovascular diseases, including stroke [19]. Although bacterial DNA has been detected in the brains of AD patients, the causal significance of these findings remains unresolved [20,21,22,23,24]. Intriguingly, recent studies suggest that microbes may persist in brain tissue even under non-inflammatory and non-traumatic conditions. By contrast, the relationship between the GM and brain tumors (BTs) is a relatively new and underexplored area. Early studies reporting altered GM profiles in BT patients provide preliminary support for a gut–brain–tumor axis [25,26,27]. Mechanistically, gut-derived metabolites—mainly short-chain fatty acids (SCFAs)—can modulate systemic immunity and BBB permeability, offering a plausible link between intestinal dysbiosis and BT pathogenesis [28].

Among primary BTs, glioblastoma (GBM) is the most aggressive and prevalent malignant glioma, characterized by rapid progression, high recurrence, and a profoundly immunosuppressive tumor microenvironment [29]. BBB disruption during gliomagenesis facilitates immune cell infiltration, which, paradoxically fosters a tumor-promoting milieu [30]. Emerging evidence suggests that GM-derived signals may amplify this CNS immunosuppression via the gut–brain axis, thereby accelerating GBM progression [31,32,33,34]. Notably, Nejman and collaborators first reported intracellular bacteria in formalin-fixed paraffin-embedded (FFPE) GBM specimens, a finding later confirmed by Zhao and colleagues [4,35].

Therefore, the aim of this study was to characterize the microbiome of both fecal and fresh brain cancerous tissue samples of BT patients, thereby reinforcing the concept of a gut–brain–tumor axis and laying the groundwork for novel diagnostic and therapeutic opportunities in neuro-oncology.

## 2. Results

### 2.1. Enrolled Patients

As detailed in Table 1, 9 patients (5 males, 4 females; mean age of 48.0 ± 13.4 years) with GBM, 18 patients with meningioma (MGM) (7 males, 11 females; mean age of 51.2 ± 13.1 years) and 12 sex- and age-matched HC volunteers (6 males, 6 females; mean age of 44.8 ± 12.7 years) were enrolled for the present study. Within the GBM group, all patients were diagnosed with grade 4 tumors, whereas in the MGM cohort, fifteen patients (83%) had grade 1 tumors and three patients (17%) were diagnosed with grade 2 tumors.

### 2.2. Gut Microbiota Composition

**Figure 1:**
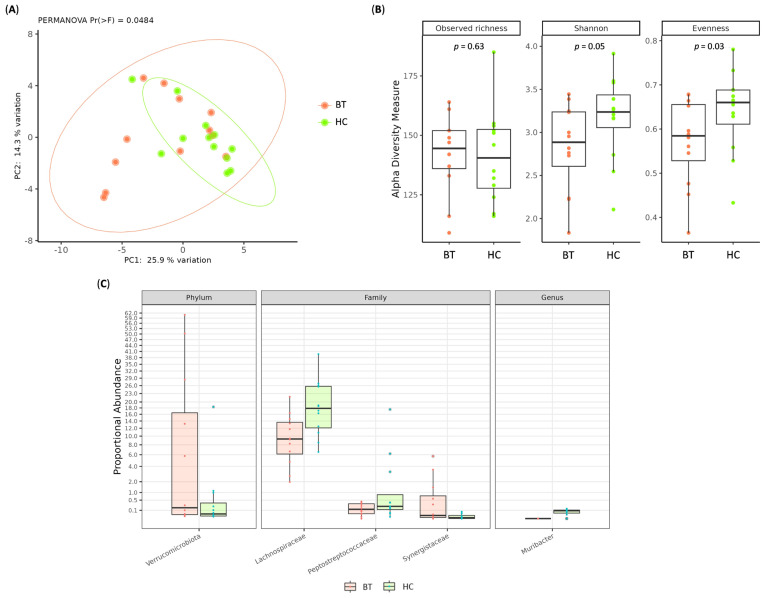
(**A**) Principal coordinate analysis (PCoA) conducted with the Hellinger distance on transformed genera abundances of stool samples among HCs and BT patients. (**B**) Box plots showing alpha diversity indices (Observed ASV, Shannon index, Pielou’s evenness) of stool samples among HCs and BT patients. (**C**) Boxplot displaying the results of differential abundances analysis of stool samples between HCs and BT patients. All results have an adjusted *p* < 0.05.

First, we evaluated whether BT patients exhibited a distinct GM composition compared to HCs. PCoA computed using the Hellinger distance on transformed genera abundances revealed a clear separation among stool samples from HCs and BT patients (PERMANOVA, *p* = 0.0484) (Figure 1A). Additionally, BT patients showed a decrease in fecal alpha diversity (Shannon index, *p* = 0.05; Evenness, *p* = 0.03) compared to HCs (Figure 1B). Furthermore, differential abundance analysis across all taxonomic levels revealed that, compared to HCs, BT patients had a significantly higher abundance of bacteria belonging to the Verrucomicrobiota phylum (log2FC = −4.0121, adj.*p* = 0.008) and the Synergistaceae family (log2FC = −6.4565, adj.*p* = 0.002), as well as lower abundances of Lachnospiraceae (log2FC = 1.3768, adj.*p* = 0.002), Peptostreptococcaceae (log2FC = 4.2549, adj.*p* = 0.002) and *Muribacter* spp. (log2FC = 10.1719, adj.*p* < 0.0001) (Figure 1C).

**Figure 2:**
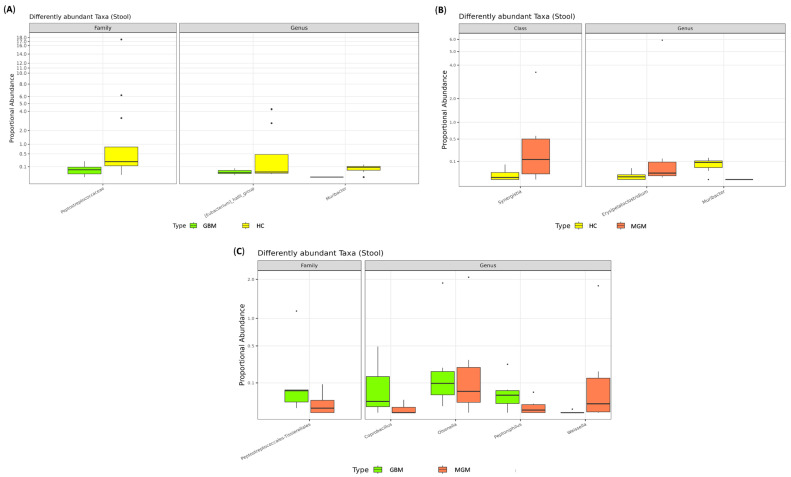
Boxplot displaying the results of differential abundances analysis between HC and GBM (**A**), HC and MGM (**B**) and GBM and MGM (**C**). All results have an adjusted. *p* < 0.05.

We then divided BT patients into GBM and MGM subgroups. Although no significant differences were observed in alpha (Figure S1A) and beta diversity indices (Figure S1B), several taxa were differentially abundant. Compared to HCs, GBM patients exhibited diminished levels of bacteria belonging to the Peptostreptococcaceae family (log2FC = −6.596, adj.*p* = 0.006) and the *Eubacterium_hallii_*group (log2FC = −6.189, adj.*p* = 0.021) and *Muribacter* (log2FC = −8.826, adj.*p* = 0.011) genera (Figure 2A). In MGM patients, increased levels of members of the Synergistia class (log2FC = 4.971, adj.*p* = 0.045) and the *Erysipelatoclostridium* genus (log2FC = 6.163, adj.*p* = 0.037), along with reduced abundance *of Muribacter* spp. (log2FC = −10.675, adj.*p* < 0.0001), were observed compared to HCs (Figure 2B). Finally, comparing GBM and MGM patients, the GBM group was enriched in members of the Peptostreptococcales–Tissierellales family (log2FC = 7.905, adj.*p* = 0.022) and the *Coprobacillus* (log2FC = 9.638, adj.*p* = 0.026), *Olsenella* (log2FC = 6.550, adj.*p* = 0.006), and *Peptoniphilus* (log2FC = 9.760, adj.*p* = 0.006) genera, alongside a reduction in *Weissella* spp. (log2FC = −11.549, adj.*p* = 0.004) (Figure 2C).

**Figure 3:**
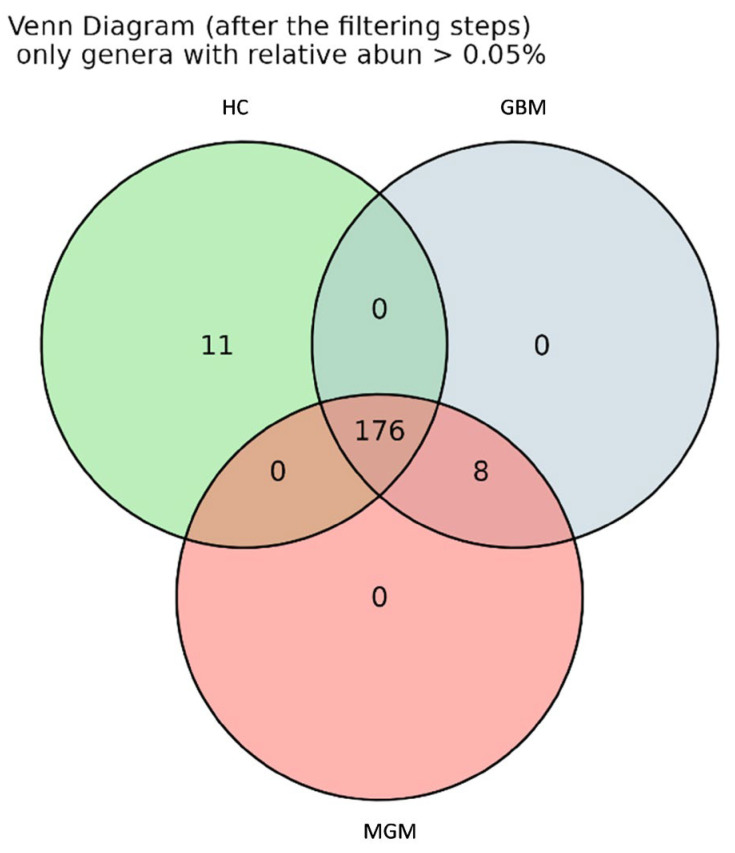
Venn diagram among HC, GBM and MGM groups including only stool genera with minimal abundance > 0.05%.

Furthermore, a Venn diagram was used to identify genera shared among the three groups (Figure 3). The analysis revealed that 176 genera were common to all groups, while 11 genera were exclusive to the HC group: *Muribacter*, *Victivallaceae*, *Prevotellaceae_UCG-004*, *Asteroleplasma*, *Anaerovibrio*, *Sarcina*, *Coriobacteriaceae_UCG-002*, *Gardnerella*, *[Bacteroides]_pectinophilus_*group, *Lachnospiraceae_UCG-007* and *Lachnospiraceae_NK3A20_*group. Notably, 8 genera, *Oligella*, *Kluyvera*, *Enterobacteriaceae*, *Weeksella*, *Dielma*, *Succiniclasticum*, *Megamonas* and *Frisingicoccus*, were shared exclusively by GBM and MGM patients, but interestingly, none were exclusive to either group.

### 2.3. Characterization of Brain Tumor Tissue Microbiome

**Figure 4:**
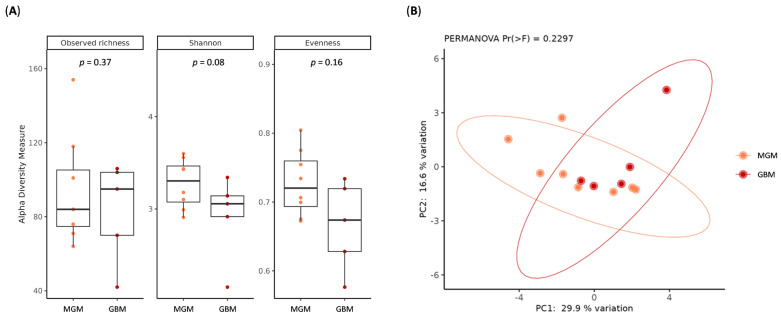
(**A**) Box plots showing alpha diversity indices (Observed ASV, Shannon index, Pielou’s evenness) of fresh BT tissue samples among MGM and GBM patients. (**B**) Principal coordinate analysis (PCoA) conducted with the Hellinger distance on transformed genera abundances of fresh BT tissue samples among MGM and GBM patients.

In addition to GM profiling, we characterized the ItM of BT tissues. In terms of microbial diversity, no statistically significant differences in alpha (Figure 4A) or beta diversity (Figure 4B) indices were observed among GBM and MGM samples.

**Figure 5:**
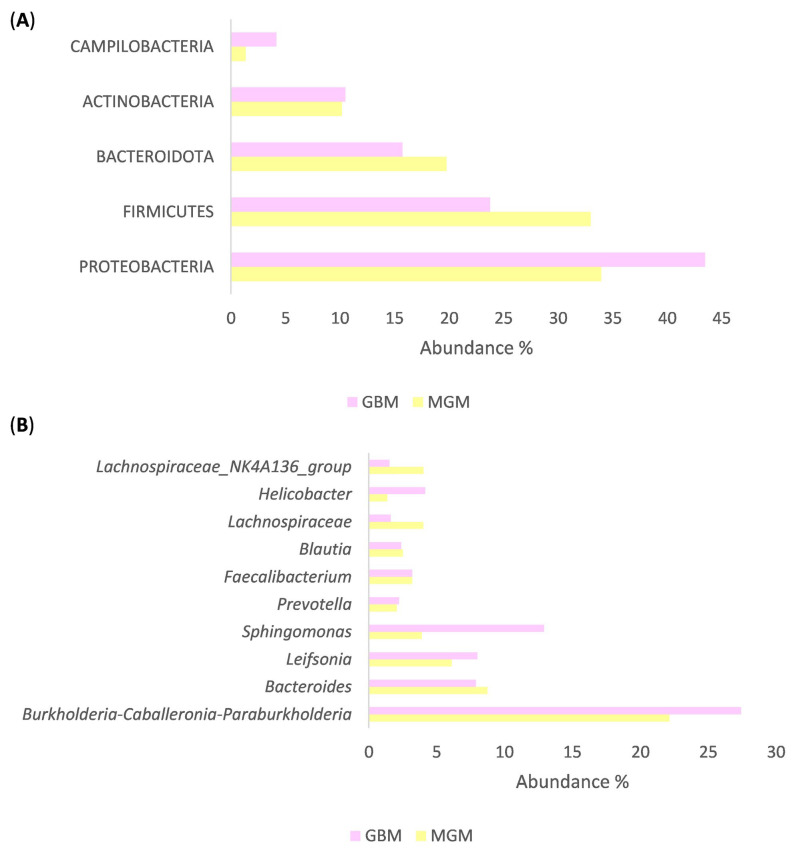
Bar chart showing global distribution of most abundant phyla (**A**) and genera (**B**) in GBM and MGM fresh BT tissues.

However, the obtained results revealed distinct profiles in microbial distributions between GBM and MGM groups. The five most abundant phyla in GBM tissues were Proteobacteria (43.49%), Bacillota (23.77%), Bacteroidota (15.73%), Actinomycetota (10.50%) and Campylobacterota (4.16%). In contrast, MGM tissues showed relative enrichment in Proteobacteria (33.9%), Bacillota (33.0%), Bacteroidota (19.78%), Actinomycetota (10.18%) and Campylobacterota (1.36%) (Figure 5A).

At the genus level, the ten most represented bacteria in GBM tissues included *Burkholderia-Caballeronia-Paraburkholderia* (27.43%), *Bacteroides* (7.89%), *Leifsonia* (8.01%), *Sphingomonas* (12.90%), *Prevotella* (2.24%), *Faecalibacterium* (3.20%), *Blautia* (2.40%), *Lachnospiraceae* (1.62%), *Helicobacter* (4.16%) and *Lachnospiraceae_NK4A136_group* (1.53%). In MGM tissues, the top genera included *Burkholderia-Caballeronia-Paraburkholderia* (22.14%), *Bacteroides* (8.75%), *Leifsonia* (6.11%), *Sphingomonas* (3.91%), *Prevotella* (2.07%), *Faecalibacterium* (3.20%), *Blautia* (2.52%), *Lachnospiraceae* (4.01%), *Helicobacter* (1.36%) and *Lachnospiraceae_NK4A136_*group (4.03%) (Figure 5B).

**Figure 6:**
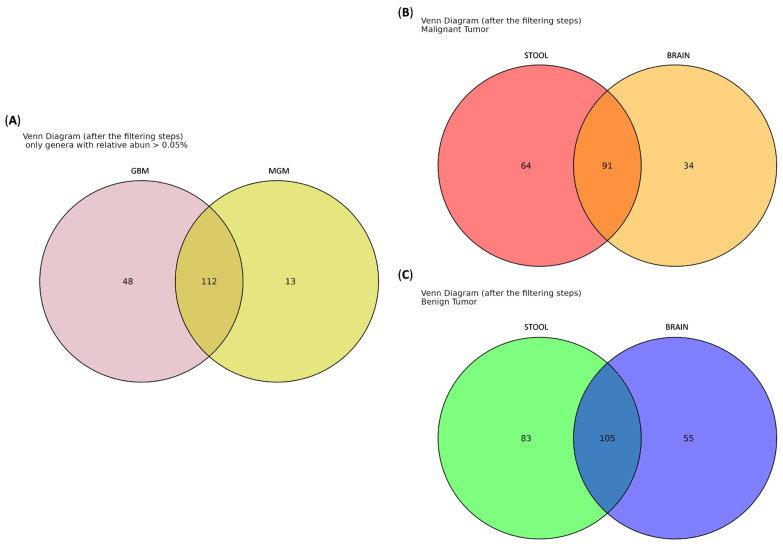
Venn diagram of fresh BT tissue among from the GBM and MGM groups, including only genera with a minimum abundance > 0.05% (**A**). Venn diagram between stool and brain samples from GBM (**B**) and MGM (**C**) cohorts including only genera with minimum abundance > 0.05%.

Although no genera reached statistical significance in differential-abundance testing, we used a Venn diagram to compare the taxa shared between GBM and MGM tissues, including only those genera with a relative abundance > 0.05% (Figure 6A). The analysis revealed that 112 genera were common to both GBM and MGM groups and, surprisingly, 48 genera were unique to the GBM tissues, while 13 genera (namely *Noviherbaspirillum*, *Mesorhizobium*, *Roseiarcus*, *Subgroup_2*, *Luteitalea*, *Candidatus_Udaeobacter*, *WPS-2*, *Candidatus_Adlerbacteria*, *Sediminibacterium*, *Porphyromonas*, *Leptotrichia*, *Melghirimyces*, *Bryobacter*) were exclusive to MGM samples.

Lastly, we generated Venn diagrams to assess the sharing of bacterial taxa between stool and tissue samples. Interestingly, in the GBM cohort, 91 genera were common to both stool and tissue samples (Table S1), while 64 genera were detected exclusively in stool and 34 were unique to brain tissue samples (Figure 6B). Similarly, in the MGM cohort, 105 genera were shared between stool and tissue samples (Table S2), with 83 genera unique to stool and 55 exclusive to brain tissue samples (Figure 6C).

## 3. Discussion

Although the CNS has traditionally been regarded as sterile and protected from microbial invasion, the discovery of a bidirectional gut–brain axis has challenged this paradigm. Over the past decade, extensive research has linked GM dysbiosis to a wide range of neurodegenerative and neuropsychiatric disorders [36,37,38]. Beyond overt infections, bacterial and fungal DNA have been detected in postmortem brain tissue from AD and PD patients, supporting the controversial but plausible presence of microbes, or their components, within both healthy and diseased brains [22,23,39,40].

In contrast, the role of the gut–brain–microbiome axis in brain tumorigenesis remains poorly defined. However, growing evidence across multiple cancer types suggests that specific bacterial taxa may influence both tumor initiation and progression [41]. To date, only a handful of studies have examined the GM of BT patients, consistently reporting significant alterations compared with healthy subjects [26,27,42,43]. In line with these findings, our analysis revealed marked differences in GM composition between BT patients and healthy individuals, suggesting that distinct microbial signatures may accompany, or even contribute to, brain cancer biology.

At the phylum level, Verrucomicrobiota, largely driven by the mucus-degrading genus *Akkermansia*, was enriched in stool samples of BT patients. This is consistent with reports linking *Akkermansia* spp. to neurological diseases such as stroke, PD and MS. Notably, both clinical and experimental studies have shown that Verrucomicrobiota and *Akkermansia* spp. abundance increases with glioma development [43,44]. In detail, Patrizz and colleagues further demonstrated in a glioma mouse model that *Akkermansia* spp. expansion decreased following treatment with temozolomide, suggesting that microbial shifts may respond to therapeutic interventions [44]. Given that *Akkermansia* spp. can erode the intestinal mucus barrier and trigger pro-inflammatory pathways, its overrepresentation may promote gut-barrier dysfunction and systemic inflammation.

An increase in *Akkemansia* spp. has also been reported in the GM of patients with type 2 diabetes and, in our cohort, 4 MGM and 1 GBM patients had this condition [45]. Intriguingly, a recent bidirectional Mendelian randomization study reported evidence for a causal relationship between type 2 diabetes and increased risk of GBM, identifying single nucleotide polymorphisms in genes involved in bone morphogenetic protein signaling and metabolic pathways that may be relevant to both conditions, though causal mechanisms remain to be elucidated [46].

Conversely, we observed depletion of SCFAs-producing taxa, particularly members of the Lachnospiraceae and Peptostreptococcaceae families. SCFA-producers are essential for maintaining gut-barrier integrity, supporting colonocyte health, and upregulating tight-junction proteins at the BBB [47]. Our findings align with prior reports: Li and colleagues documented a reduction in Lachnospiraceae in BT patients compared with healthy subjects [17], while a Mendelian randomization study suggested a protective effect of Peptostreptococcaceae against glioma [43]. Taken together, the loss of these SCFA-producing taxa likely reflects an imbalance in GM composition that compromises integrity of both intestinal and brain barriers. Increased BBB permeability may, in turn, promote tumor progression by allowing greater systemic influence on the CNS microenvironment.

When stratifying BT samples into GBM and MGM groups, we observed no significant differences in global fecal α- or β-diversity, consistent with prior reports that overall microbial richness and community composition remain similar across benign and malignant BTs [26]. However, several taxa differed markedly between GBM and MGM patients, suggesting that microbial signatures may correlate with tumor malignancy rather than community-wide shifts. In GBM patients, we identified significant depletions in members of the Peptostreptococcaceae family, as well as in the *Eubacterium_hallii_*group and *Muribacter* genera. *Eubacterium hallii*, a butyrate- and propionate-producer within the Lachnospiraceae family, is known to reinforce epithelial barriers and modulate immune responses, thereby supporting gut–host homeostasis [48,49]. Conversely, MGM patients showed enrichment of taxa in the Synergistia class and the *Erysipelatoclostridium* genus, alongside a similar decrease in *Muribacter* spp. Direct comparisons between GBM and MGM groups revealed distinct microbial signatures: GBM samples were significantly enriched in *Peptostreptococcales-Tissierellales*, *Coprobacillus*, *Olsenella*, and *Peptoniphilus* genera, whereas *Weissella* spp. were depleted. Interestingly, in contrast to our results, *Olsenella* spp. and *Coprobacillus* spp. have recently been implicated in glioma protection [43]. For instance, *Olsenella* species produce the purine metabolite inosine, which exerts neuroprotective and anti-inflammatory effects by promoting M2 microglial polarization and tissue repair [50,51,52]. However, this enrichment may instead reflect a tumor-driven strategy to subvert immune regulation, whereby polarization toward the M2 phenotype fosters an immunosuppressive microenvironment that promotes tumor growth and enables immune evasion, a mechanism frequently observed in diverse cancer types [53,54].

Across all cohorts, we identified 176 shared genera, as well as taxa uniquely present in HCs or BT patients. 11 genera were exclusive to HCs, including *Muribacter*, *Victivallaceae*, *Prevotellaceae_UCG-004*, *Asteroleplasma*, *Anaerovibrio*, *Sarcina*, *Coriobacteriaceae_UCG-002*, *Gardnerella*, *[Bacteroides]_pectinophilus_*group, *Lachnospiraceae_UCG-007*, and *Lachnospiraceae_NK3A20_*group, suggesting potential roles in maintaining gut homeostasis. By contrast, 8 genera (i.e., *Oligella*, *Kluyvera*, *Enterobacteriaceae*, *Weeksella*, *Dielma*, *Succiniclasticum*, *Megamonas* and *Frisingicoccus*) were detected exclusively in BT patients, though without specificity for GBM or MGM. These distinct microbial signatures highlight potential biomarkers of BT-associated dysbiosis and align with earlier proposals for their use as non-invasive diagnostic tools or therapeutic targets [27].

In addition to profiling the fecal microbiome, we characterized the ItM of snap-frozen BT tissues of the same patient cohort. To our knowledge, this study is among the first to directly assess the bacterial landscape of fresh BT samples. Beyond oncology, direct bacterial–CNS interactions have been reported in AD, PD, MS, ALS and Huntington’s disease, supporting a broader role for microbes in CNS pathology [40,55].

Our results revealed that GBM and MGM tissues harbored similar dominant phyla but with notable quantitative differences: GBM samples were enriched in Proteobacteria, Actinomycetota, and Campylobacterota, whereas MGM tissues were richer in Bacillota and Bacteroidota. These phylum-level distributions are consistent with recent findings indicating that approximately 90% of cancerous and adjacent healthy brain tissues dominated by Bacillota and Proteobacteria [56]. Regarding overall community structure, no significant differences were observed in α- or β-diversity, suggesting that, as in the GM, malignancy-associated effects are more evident at finer taxonomic levels.

At the genus level, GBM tissues were enriched in *Burkholderia–Caballeronia–Paraburkholderia*, *Leifsonia*, *Sphingomonas*, *Prevotella*, *Faecalibacterium*, and *Helicobacter*, while MGM samples showed higher levels of *Bacteroides* and *Blautia*. These differences likely reflect tumor-specific microenvironmental factors, such as hypoxia, metabolic reprogramming, and immune modulation, that selectively favor colonization by particular microbes. Notably, members of the Burkholderiaceae family have previously been detected in AD brain tissue, and in breast cancer–bearing mice undergoing chemotherapy, their abundance correlated positively with pro-inflammatory mediators and negatively with the expression of gut tight-junction proteins [57,58]. Similarly, the *Helicobacter* genus—best represented by *H. pylori*, a well-established gastric carcinogen—was more abundant in GBM tissues. *H. pylori* releases pro-inflammatory cytokines (e.g., TNF-α, IL-6) and virulence factors (VacA, HP-NAP) that activate microglia, promote neuroinflammation, and increase blood–brain barrier permeability, mechanisms that may exacerbate GBM malignancy [59]. Conversely, *Blautia* emerged as a discriminative genus in non-GBM patients. This genus has been previously reported as a marker distinguishing PD patient from controls and has been associated with host brain genes involved in energy metabolism, proteins degradation, and mitochondrial function [60]. Remarkably, a recent study by Sipos et al., identified *Blautia* as the prevalent genus in tumor-adjacent tissue and tumor tissue of BT patients [56].

Furthermore, Venn diagram analysis revealed a shared core microbiome of 112 genera across GBM and MGM patients, with 48 genera unique to GBM and 13 exclusive to the MGM cohort.

Among these 13 MGM–specific genera, *Porphyromonas* was notably detected in brain tissue. The oral pathogen *P. gingivalis* has been anti-correlated with glioma grade: Wen et al. reported that *Porphyromonas* species were less abundant in the oral microbiota of patients with high-grade glioma than in those with low-grade glioma or healthy subjects [61]. Epidemiological evidence further supports this link, as glioma patients in China have been shown to exhibit a higher prevalence of periodontitis, and by extension, *P. gingivalis* colonization, compared with patients with benign BTs [62]. Mechanistically, *P. gingivalis* virulence factors, including outer membrane vesicles, LPS, and gingipains, can disrupt BBB integrity, facilitating bacterial translocation into CNS tissue, while its LPS can also exert pro-tumorigenic effects [63]. In addition, both *Leptotrichia* and *Porphyromonas* display a negative association with glioma grade, consistent with our observations in MGM brain tissue [61]. Collectively, these findings suggest that *Porphyromonas* and *Leptotrichia* may serve as negative biomarkers of glioma malignancy, potentially reflecting oral–brain microbial crosstalk that influences tumor behavior.

Finally, our Venn analysis comparing stool and tumor tissue microbiome supports the hypothesis of a bidirectional gut–brain microbial interaction and reveals both shared and niche-adapted communities in GBM and MGM patients. A substantial set of shared genera (91 in GBM; 105 in MGM) suggests that many gut-resident taxa can also inhabit the tumor microenvironment, potentially via translocation across a compromised BBB or through systemic circulation of microbial metabolites and components. These shared taxa may act as reservoirs for cancer-associated microbes or represent host-wide microbial signatures detectable in both stool and tissue. In contrast, the presence of tissue-exclusive genera (34 in GBM; 55 in MGM) is particularly intriguing. These taxa may harbor unique adaptations that permit survival within the tumor niche or represent low-abundance gut microbes that become enriched in the brain under selective pressures such as hypoxia, altered pH, immune modulation, hallmarks of gliomas and meningiomas. Notably, GBM harbored fewer tissue-exclusive genera compared to MGM, perhaps reflecting its more aggressive biology and dependence on a narrower set of highly specialized microbes. Conversely, benign meningiomas may provide a less hostile microenvironment that supports a broader spectrum of colonizers. Tumor hypoxia represents a well-recognized feature of glioma progression. Li and colleagues reported that glioma tissues were enriched in anaerobic genera, underscoring the potential biological relevance of such selective pressure. Moreover, their study observed significant differences between the microbial composition of fecal and tumor samples from the same glioma cohort, suggesting that the GM may not represent the sole source of intratumoral bacteria [26].

Overall, despite certain limitations such as the observational design, modest patient cohort, and the absence of non-tumor brain tissues as controls (due to ethical reasons), this study provides an innovative report encompassing several novel aspects: (i) the characterization of the ItM using fresh brain tissue samples, (ii) the comparison of ItM profiles between GBM and MGM, (iii) the correlation between GM and ItM in the same BT patients, and (iv) important implications for the clinical management of BT patients.

Future in vivo and in vitro studies should aim to elucidate the mechanistic roles of gut–brain microbial interactions in tumor biology and to evaluate their potential as diagnostic biomarkers or therapeutic targets in human brain cancers.

## 4. Materials and Methods

### 4.1. Patients’ Enrolment

A total of 27 patients with histologically confirmed primary BTs were enrolled at Frantz Fanon University Hospital (Blida, Algeria) and Khelil Amrane Hospital (Bejaia, Algeria). Exclusion criteria included prior radiotherapy or chemotherapy, as well as the use of antibiotics, probiotics, prebiotics, or symbiotics within three months before enrollment. In addition, 12 sex- and age-matched healthy controls (HCs) were recruited. Fresh stool and brain tissue samples were collected at the time of surgery, or provided (stool) by HCs, and immediately stored at −80 °C until further analyses. All study procedures adhered to the Declaration of Helsinki, and written informed consent was obtained from each participant. The protocol was approved by the Ethics and Deontology committee of the university of Bejaia (Ref. 03/C.E.D/UB/2023).

### 4.2. Fecal and Brain Microbiota Characterization

Genomic DNA was extracted from stool samples using the DNeasy PowerLyzer PowerSoil Kit (Qiagen, Hilden, Germany) and from fresh brain tissues using the DNeasy Blood and Tissue Kit (Qiagen, Hilden, Germany). DNA quality and concentration were assessed with a NanoDrop ND-1000 spectrophotometer (Thermo Fisher Scientific, Waltham, MA, USA) and a Qubit Fluorometer (Thermo Fisher Scientific, Waltham, MA, USA) before being stored at −20 °C. Total DNA samples were subsequently sent to IGA Technology Services (Udine, Italy), where amplicons of the variable V3–V4 region of the bacterial 16S rRNA gene were sequenced in paired-end (2 × 300 cycles) on the Illumina MiSeq platform (San Diego, CA, USA), according to the Illumina 16S Metagenomic Sequencing Library Preparation protocol. Two negative control samples were included in parallel throughout the DNA extraction and amplification process to monitor potential contamination; sequencing of these controls yielded no detectable amplicon sequence variants (ASVs), supporting the absence of exogenous DNA contamination. Afterwards, demultiplexed reads were processed using QIIME2 (v.2022.8). Sequencing primers and reads lacking primers were removed using Cutadapt (v.3.4). DADA2 was employed for quality filtering, paired-end read merging and chimera removal, after trimming low-quality nucleotides from both forward and reverse reads. ASVs were generated, and taxonomic assignments were performed through the Scikit-learn multinomial naive Bayes classifier re-trained on the SILVA database (release 138) for the V3–V4 region. To enhance the accuracy of downstream analyses, cross-amplified host DNA was filtered by aligning ASVs against the GRCh38 human reference genome using Bowtie2 (v.2.2.5) [64,65]. ASVs assigned to Chloroplast or Mitochondria, according to the SILVA database, were removed. Additionally, ASVs assigned to genera with an average relative abundance below 0.01% cut-off or present in only one sample across the dataset were excluded [66,67].

### 4.3. Statistical Analysis

Statistical analyses of bacterial communities were conducted in R (v.4.3) using the packages phyloseq (v.1.44.0), vegan (v.2.6-4), DESeq2 (v.1.40.1) and other packages satisfying their dependencies. Data visualization was performed using ggplot2 (v.3.4.2), ggvenn (v.0.1.9), ggh4x (v.0.2.4) and ggpubr (v.0.40) packages. The observed richness and Shannon indices were used to estimate the alpha-diversity in each sample using the function estimate_richness from phyloseq. Pielou’s evenness index was calculated using the formula E = S/log(R), where S is the Shannon diversity. The “core” microbiota—defined as genera with >0.05%. relative abundance in at least three samples—was visualized with a Venn diagram. Principal coordinate analysis (PCoA) was performed using the Hellinger distance on transformed genera abundances. PERMANOVA and Betadisper were used to test the statistical significance of the beta-diversity distances and dispersions, respectively. Differential abundance analyses at various taxonomic ranks were performed on raw count data with DESeq2. To reduce noise, taxa with a DESeq2 baseMean < 50 were excluded from the results regardless of their statistical significance. *p*-values were adjusted using the Benjamini–Hochberg method, and results with an adjusted *p*-value < 0.05 were considered significant. 

## Figures and Tables

**Table 1:** Clinical and demographic characteristics of enrolled brain tumor patients and healthy controls.

	Glioblastoma	Meningioma	Healthy Controls
**Subjects**	n = 9	n = 18	n = 12
**Age (mean ± SD)**	48.0 ± 13.4	51.2 ± 13.1	44.8 ± 12.7
**Gender** Male Female	5 4	7 11	6 6
**Other pathology** Diabetes Hypertension	1 2	4 5	- -
**Tumor grade** Grade 1 Grade 2 Grade 3 Grade 4	9	15 3	- - - -

## Data Availability

The data presented in this study are deposited in the NCBI Gene Expression Omnibus (GEO) repository, accession number GSE303995.

## Supplementary Materials

The supporting information can be downloaded at: https://www.mdpi.com/article/10.3390/ijms262311290/s1.
