# Supplementary material for: Specific Intratumoral Microbiome Signatures in Human Glioblastoma and Meningioma: Evidence for a Gut–Brain Microbial Axis
Source: Int J Mol Sci. 2025 Nov 22;26(23):11290. doi: 10.3390/ijms262311290 (PMC12692222; doi:10.3390/ijms262311290)
Supplement: Supplementary file 1 [file ijms-26-11290-s001.zip › ijms-3947773-supplementary.pdf]

SUPPLEMENTARY MATERIALS

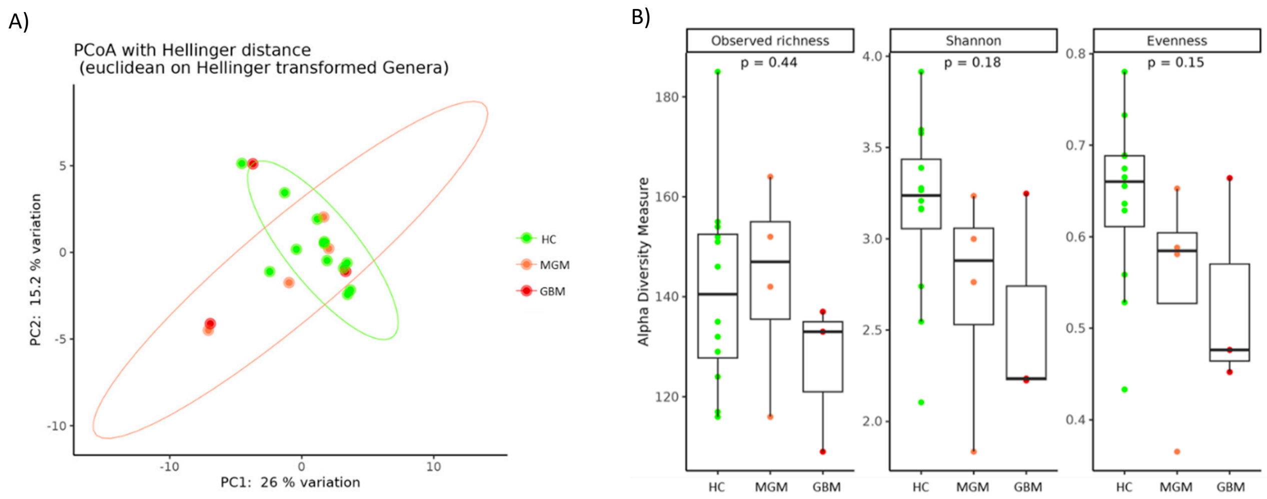

Figure S1. (**A**) Principal coordinate analysis (PCoA) conducted with the Hellinger distance on transformed genera abundances of stool samples among HC and both GBM and not MGM patients. (**B**) Box plots showing alpha diversity indices (Observed ASV, Shannon index, Pielou's evenness) of stool samples among HC and both GBM and MGM patients.

SUPPLEMENTARY TABLES

The 91 genera shared among stool and brain tissue samples in GBM patients
Burkholderia-Caballeronia-Paraburkholderia, Bacteroides, Sphingomonas, Escherichia-Shigella, Akkermansia, Leifsonia, Prevotella, Faecalibacterium, Helicobacter, UCG-002, Blautia, [Eubacterium]_coprostanoligenes_group, Christensenellaceae_R-7_group, Subdoligranulum, Dialister, Lachnospiraceae_NK4A136_group, Alistipes, NK4A214_group, Bifidobacterium, Coprococcus, Megasphaera, Parabacteroides, Collinsella, Muribaculaceae, Odoribacter, Cloacibacillus, Ruminococcus, Lactobacillus, [Ruminococcus]_torques_group, Streptococcus, Megamonas, Roseburia, Agathobacter, Klebsiella, Lachnoclostridium, Dorea, Succinivibrio, Prevotellaceae_UCG-001, Desulfovibrio, Clostridia_vadinBB60_group, Catenibacterium, Phascolarctobacterium, Fusicatenibacter, Romboutsia, Sutterella, Lachnospira, Veillonella, Pseudomonas, UCG-005, [Eubacterium]_hallii_group, Acidaminococcus, Alloprevotella, Anaerostipes, Monoglobus, Butyricicoccus, UCG-003, Barnesiella, Clostridium_sensu_stricto_1, Oscillibacter, Butyricimonas, [Ruminococcus]_gnavus_group, Holdemanella, Lachnospiraceae_ND3007_group, Paraprevotella, UBA1819, Rikenellaceae_RC9_gut_group, Clostridia_UCG-014, [Clostridium]_innocuum_group, Flavonifractor, Porphyromonas, Eubacterium]_siraeum_group, Incertae_Sedis, Erysipelotrichaceae_UCG-003, Enterococcus, Bilophila, Turicibacter, UCG-010, Enterorhabdus, Parasutterella, Lachnospiraceae_UCG-004, [Ruminococcus]_gauvreauii_group, GCA-900066575, Lachnospiraceae_UCG-010, Erysipelatoclostridium, Intestinibacter, Methanobrevibacter, Senegalimassilia, Gastranaerophilales, Tyzzerella, [Eubacterium]_xylanophilum_group, Coprobacillus

Table S1. Bacterial genera shared among stool and brain tissue samples in GBM patients, ranked in descending order based on their relative abundance.

The 105 genera shared among stool and brain tissue samples in MGM patients
Burkholderia-Caballeronia-Paraburkholderia, Akkermansia, Escherichia-Shigella, Bacteroides, Faecalibacterium, Subdoligranulum, UCG-002, Leifsonia, Lachnospiraceae_NK4A136_group, Bifidobacterium, Christensenellaceae_R-7_group, Blautia, [Eubacterium]_coprostanoligenes_group, Sphingomonas, Clostridia_UCG-014, Dialister, Muribaculaceae, Prevotella, CAG-352, Alistipes, Collinsella, Ruminococcus, UCG-005, Lactobacillus, Parabacteroides, Coprococcus, Agathobacter, NK4A214_group, Catenibacterium, Methanobrevibacter, Dorea, [Ruminococcus]_torques_group, Helicobacter, Streptococcus, Veillonella, Roseburia, Holdemanella, Klebsiella, Odoribacter, Clostridia_vadinBB60_group, Anaerostipes, Erysipelatoclostridium, Prevotellaceae_UCG-001, Monoglobus, Lachnoclostridium, Romboutsia Barnesiella, Butyricicoccus, Cloacibacillus, [Eubacterium]_eligens_group, Lachnospira, Fusicatenibacter, Phascolarctobacterium, [Ruminococcus]_gnavus_group, [Eubacterium]_xylanophilum_group, Colidextribacter, Prevotellaceae_NK3B31_group, [Eubacterium]_hallii_group, Megasphaera, Clostridium_sensu_stricto_1, Incertae_Sedis, UBA1819, [Eubacterium]_siraeum_group, Desulfovibrio, Erysipelotrichaceae_UCG-003, Rikenellaceae_RC9_gut_group, Sutterella, Oscillibacter, Paraprevotella, Alloprevotella, Enterorhabdus, Succinivibrio, UCG-010, [Eubacterium]_ruminantium_group, Lachnospiraceae_ND3007_group, Intestinibacter, Senegalimassilia, Parasutterella, UCG-003, Enterococcus, Pseudomonas, [Ruminococcus]_gauvreauii_group, Lachnospiraceae_UCG-001, Flavonifractor, Tyzzerella, Bilophila, Coprobacillus, Haemophilus, Intestinimonas, GCA-900066575, Lachnospiraceae_FCS020_group, Butyricimonas, [Clostridium]_innocuum_group, Asteroleplasma, Turicibacter, Fusobacterium, [Eubacterium]_ventriosum_group, Marvinbryantia, Gastranaerophilales, Elusimicrobium, Acidaminococcus, [Bacteroides]_pectinophilus_group, Gemella, Lachnospiraceae_UCG-004, Lachnospiraceae_UCG-010

Table S2. Bacterial genera shared among stool and brain tissue samples in MGM patients, ranked in descending order based on their relative abundance.
